# Identification and characterization of *Phaseolus* *vulgaris* endornavirus 1, 2 and 3 in common bean cultivars of East Africa

**DOI:** 10.1007/s11262-023-02026-7

**Published:** 2023-08-10

**Authors:** Thomas J. Brine, Sam Crawshaw, Alex M. Murphy, Adrienne E. Pate, John P. Carr, Francis O. Wamonje

**Affiliations:** 1 Department of Plant Sciences, University of Cambridge, Cambridge, CB2 3EA UK; 2 Pest and Pathogen Ecology, National Institute of Agricultural Botany, East Malling, ME19 6BJ UK

**Keywords:** Endornaviruses, *Phaseolusvulgaris*, Mixed virus infections

## Abstract

Persistent viruses include members of the family *Endornavirus* that cause no apparent disease and are transmitted exclusively via seed or pollen. It is speculated that these RNA viruses may be mutualists that enhance plant resilience to biotic and abiotic stresses. Using reverse transcription coupled polymerase chain reactions, we investigated if common bean (*Phaseolus vulgaris* L.) varieties popular in east Africa were hosts for *Phaseolus* *vulgaris* endornavirus (PvEV) 1, 2 or 3. Out of 26 bean varieties examined, four were infected with PvEV1, three were infected with both PvEV1 and PvEV2 and three had infections of all three (PvEV) 1, 2 and 3. Notably, this was the first identification of PvEV3 in common bean from Africa. Using high-throughput sequencing of two east African bean varieties (KK022 and KK072), we confirmed the presence of these viruses and generated their genomes. Intra- and inter-species sequence comparisons of these genomes with comparator sequences from GenBank revealed clear species demarcation. In addition, phylogenetic analyses based on sequences generated from the helicase domains showed that geographical distribution does not correlate to genetic relatedness or the occurrence of endornaviruses. These findings are an important first step towards future investigations to determine if these viruses engender positive effects in common bean, a vital crop in east Africa.

**Supplementary Information:**

The online version contains supplementary material available at 10.1007/s11262-023-02026-7.

## Introduction

The so-called plant ‘persistent viruses’ (PVs) do not typically cause disease and are exclusively vertically inherited. Examples of PVs have been found in at least five ICTV-recognized families, specifically, the *Amalgaviridae, Chrysoviridae, Endornaviridae*, *Partitiviridae* and *Totiviridae* [1–3]*. Endornaviridae* are classified into two genera: *Alphaendornavirus*, members of which infect plants, fungi and oomycetes, and *Betaendornavirus*, members of which have been found only in ascomycete fungi [4]. The endornaviruses *Phaseolus* *vulgaris* endornavirus (PvEV) 1, PvEV2 and PvEV3 that occur in some lines of common bean (*Phaseolus vulgaris*) are assigned to the *Alphaendornavirus* genus by the ICTV [5, 6].

Endornavirus genomic RNAs vary in length between 9.6 and 17.6 kb and accumulate in planta as double-stranded RNA molecules associated with the viral RNA-dependent RNA polymerase (RdRp) [5, 7, 8]. However, they are now thought to be positive-sense single-stranded RNA viruses that accumulate predominantly in the form of the double-stranded replicative intermediate [9, 10]. The single open reading frame of an endornavirus is inferred to encode a polyprotein, which contains the RdRp sequence in the C-terminal domain, together with other putative functional domains encoded by some but not all endornaviruses [11–13]. These additional domains include sequences with homology to a viral RNA helicase, to a bacterial capsular polysaccharide synthase, to a UDP-glycosyltransferase, and a methyltransferase [7]. Endornavirus genomes do not encode a coat protein and, in contrast to other PVs, these viruses do not form true virions [7].

Endornaviruses and other PVs occur widely amongst wild plants and in crops, including common bean, pepper, rice and barley [12, 14–17]. The presence of PVs in many wild and cultivated plants is consistent with the theory that endornavirus infection confers a benefit to the host plant [18]. For example, the output of plant volatile organic compounds is altered in bell pepper (*Capsicum annuum*) plants infected with the partitivirus pepper cryptic virus 1 and this repels aphids (*Myzus persicae*). This is thought to protect plants from aphid damage and may provide indirect protection from infection by aphid-transmitted pathogenic viruses [19]. Also, it has been shown that plants of North American common bean varieties harbouring PvEV1 and PvEV2 yielded longer pods and a greater mass of seeds than those without [20]. Furthermore, when a coat protein gene sequence of the white clover cryptic virus (WCCV) isolated from a *Trifolium repens* (white clover) cDNA library was expressed in *Lotus japonicus* plants, formation of nitrogen-fixing root nodules by symbiotic bacteria was inhibited in an abscisic acid signalling-dependent manner. It was inferred that in white clover, WCCV plays a beneficial role in preventing the overproduction of nodules [21]. However, there are counterexamples. For example, an inherited double-stranded RNA in a line of broad bean (*Vicia faba*), later identified as an endornavirus, was associated with a male sterility phenotype [11]. More recently, the persistent virus southern tomato virus (STV: a double-stranded RNA virus classified in the *Amalgaviridae*) was suggested to influence the interactions of tomato (*Solanum lycopersicum*) with the infectious viruses pepino mosaic virus (PepMV) and cucumber mosaic virus (CMV) [22]. It was contended that the presence of STV increased the titre of CMV transiently in the early stages of infection and data suggested that STV increases the severity of synergistic symptoms caused by CMV-PepMV coinfection [22]. However, STV also enhances seed production in tomato, i.e. a beneficial effect [23]. Indeed, on balance, most of the literature on the effects of PVs on plant fitness suggest that these viruses have neutral or beneficial effects.

Common bean originated in central Mexico and was domesticated in Mesoamerica and the Andean region of South America [24]. In east and central Africa, common bean provides the second and third most important sources of dietary protein and calories, respectively, for over 100 million people [25, 26]. Bean’s zinc and iron content help alleviate anaemia, which affects approximately 50% of children < 5 years old, 25% of children 5–15 years old and 30% of women [27]. Additionally, common bean plant roots harbour nitrogen-fixing bacteria and when grown as intercrops with staples, such as maize, cassava and banana, bean plants enrich soils with nitrogen. Despite the release of many improved common bean varieties, productivity is constrained by pests and diseases, including insect-transmitted viruses [28]. New means to increase the resilience of common bean to such attacks are needed, and PVs such as bean endornaviruses may in the future help with this. To further our understanding of endornavirus occurrence in common bean in east and central Africa crop species, we screened for PvEV1, PvEV2 and PvEV 3 in 26 bean cultivars grown in Kenya, Rwanda and Uganda. The presence of PvEV1 or PvEV2 in common bean is determined, to some extent, by a variety’s pedigree, with PvEV1 and PvEV2 more common in cultivars originating in Mesoamerica than in the Andes [29]. Indeed, a starting hypothesis for this work was that the complement of endornaviruses carried by a common bean line might reflect which breeding programme gave rise to the line. In this study, we detected PvEV1, PvEV2 and PvEV3. Though PvEV1 and PvEV2 have been detected in African common bean cultivars before [30, 31], our detection of PvEV3 in some varieties is a first for Africa.

## Materials and methods

### Plant growth, nucleic acid extraction and reverse transcription coupled polymerase chain reaction (RT-PCR) analyses

Seeds of 26 different common bean varieties were sourced through the bean research programmes of the Kenya Agricultural and Livestock Research Organization (KALRO) in Kenya and the International Centre for Tropical Agriculture (CIAT) in Uganda. The ultimate provenances of the different varieties, where known, were noted (Table 1). Bean seeds were germinated at 25 °C for 5 days in a Petri dish lined with moistened filter paper similar to methods described earlier for growing bean plants [32]. Once germinated, the radicle was excised, and total RNA was extracted using the Norgen Total RNA kit (Norgen Biotek, Thorold, Ontario, Canada). The quality and quantity of RNA were determined using a Nanodrop spectrophotometer (Thermo Scientific, Waltham, MA, USA). RNA was reverse transcribed with GoScript™ (Promega, Madison, Wisconsin, USA) following the manufacturer’s instructions to obtain the templates for polymerase chains reactions (PCRs) using previously described methods [32].

**Table 1:** Detection of PvEV1, 2 and 3 in 26 cultivars of common bean

Line	Origin	PvEV1	PvEV2	PvEV3
Cultivars with no PvEVs detected				
KANYEBWA	CIAT—Uganda	−	−	−
MASINDI YELLOW	CIAT—Uganda	−	−	−
CAB2	CIAT—Rwanda	−	−	−
CAB96	CIAT—Rwanda	−	−	−
MAC44	CIAT—Rwanda	−	−	−
GLP585 (Wairimu Dwarf)	KALRO—Thika, Kenya	−	−	−
GLP2 (Rosecoco)	KALRO—Thika, Kenya	−	−	−
GLP24 (Canadian Wonder)	KALRO—Thika, Kenya	−	−	−
GLP92 (Mwitemania)	KALRO—Thika, Kenya	−	−	−
KATB1	KALRO—Katumani, Kenya	−	−	−
KATB9	KALRO—Katumani, Kenya	−	−	−
KATX56	KALRO—Katumani, Kenya	−	−	−
GASILIDA	RAB—Kigali, Rwanda	−	−	−
RWV1129	RAB—Kigali, Rwanda	−	−	−
RWV3316	RAB—Kigali, Rwanda	−	−	−
BLACK VALENTINE	USA (commercially sourced)	−	−	−
Cultivars with PvEV1				
G2333^a^	CIAT	+	−	−
RED40	KALRO—Kakamega, Kenya	+	−	−
GLP1127	KALRO—Thika, Kenya	+	−	−
RWR1668	RAB—Kigali, Rwanda	+	−	−
Cultivars with PvEV1 and PvEV2				
KK022	KALRO—Kakamega, Kenya	+	+	−
RWR2245	RAB—Kigali, Rwanda	+	+	−
SER16	RAB—Kigali, Rwanda	+	+	−
Cultivars with PvEV1, PvEV2 and PvEV3				
MCM2001	CIAT—Uganda	+	+	+
KK072	KALRO—Kakamega, Kenya	+	+	+
RWR2075	RAB—Kigali, Rwanda	+	+	+

Identification of endornavirus RNA using RT-PCR is indicated by +, and − indicates no endornavirus present

*RAB* Rwanda Agriculture Board, *KALRO* Kenya Agriculture and Livestock Research Organization, *CIAT* Centro Internacional de Agricultura Tropical/International Center for Tropical Agriculture

^a^G2333 is a Mexican landrace [46] that has been distributed internationally and assigned different names in Rwanda (Umubano), Kenya and Tanzania (Lyamungu 85), and Uganda (NABE10)

Initial PCR was done using previously described primers targeting the Helicase and RdRp domains and using methods already described and a decision taken to proceed using the helicase gene sequences [31]. Further, two primer pairs for PvEV2 and PvEV3 helicase regions were designed using SnapGene and the NCBI primer design tool. The sequences used to design primers were PvEV2—GenBank No: AB719398.1 and for PvEV3—GenBank No: NC_040558.1. Details of all primers used and expected product size are provided in the supplementary information (Table S1). PCR was done using the BioMix Red (Bioline, Hessel, UK) reagent mix, and PCR products were resolved electrophoretically using a 1% w/v agarose gel stained with ethidium bromide and visualized in a gel documentation system. Amplicons of the expected product size were excised, purified, diluted to the appropriate concentration and submitted for semi-automated Sanger sequencing [33, 34] at Source BioScience, UK Ltd (Cambridge, UK).

### High-throughput sequencing (HTS) for endornaviruses

RNA was extracted from germinated seeds of the cultivars KK022 and KK072 (from which RT-PCR detected PvEV) using Norgen’s Total RNA kit (Norgen Biotek, Thorold, Ontario, Canada) and used to make sequencing libraries with the Zymo-Seq RiboFree Total RNA Library Kit. Libraries were sequenced on the Illumina NextSeq sequencer (Illumina, San Diego, USA) at Cambridge Genomic Services, Pathology Department, Cambridge University. Bioinformatic analyses for de novo virus detection were done using VirFind with default settings using the FastQ files as the input [35]. In summary, FastQ files for KK072 and KK022 were uploaded to the VirFind ftp server. Files were converted to Fasta format, and de novo sequence assembly done by Trinity [36] and SPAdes [37]. The assembled contigs were subjected to Basic Local Alignment Search Tool (BLAST) [38] analysis. Specifically, the BLASTn database was used, with the e-value left at the fault 0.01. This analysis annotated contigs that were the most likely ‘virus’ hits.

To generate whole-genome information, paired-end reads were bioinformatically analysed using the Geneious Prime 2020 version (Biomatters, Auckland, New Zealand). For each sample, the paired-end reads generated from Illumina sequencing were merged using the BB Merge function [39] and then mapped to reference genomes from GenBank using Geneious read mapper, all implemented in Geneious Prime. For PvEV1, reads from KK022 and KK072 were mapped to PvEV1 sequence MF375892. PVEV2 reads from KK022 and KK072 were mapped to a PvEV isolate AB719398. Lastly, for PvEV3, reads from KK072 were mapped to PvEV3 sequence NC_040558. Contigs mapping to the reference genomes were used to generate consensus sequences for endornaviruses from each of the samples using the Geneious Prime software.

### Sequence analysis and phylogenetics

BLASTn (https://blast.ncbi.nlm.nih.gov/Blast.cgi) searches were performed for each of the Sanger generated sequences to confirm the virus nature of the sequences and to obtain corresponding sequences to include in the phylogenetic analysis. Appropriate comparator sequences for the helicase domains of other endornaviruses were downloaded from GenBank. Sequence alignments were conducted in Muscle alignment tool in MEGA X software [40]. The Maximum Likelihood model was used to construct the phylogenetic trees using the GTR + G + I model determined in the ‘Find best’ model (ML) option in MEGA 11 [40, 41]. In all phylogenies, node significance was evaluated with 1000 bootstrap replications. Pairwise sequence comparisons were made using the Sequence Demarcation Tool software [42].

For the five near-complete genomes, which were consensus sequences from mapping in Geneious Prime, phylogenetic analyses were conducted using appropriate comparator sequences downloaded from GenBank using methods similar to those described above. Additionally, the sequences were subjected to a search for predicted open reading frames (ORFs) for annotation using the ORF finder tool (https://www.ncbi.nlm.nih.gov/orffinder/).

The putative polyprotein sequences encoded by the ORFs were further analysed for putative domains by querying the conserved domains search database in NCBI (https://www.ncbi.nlm.nih.gov/Structure/cdd/wrpsb.cgi?).

## Results

### Screening for *Phaseolus vulgaris* endornaviruses 1, 2 and 3 in bean varieties from Kenya, Rwanda and Uganda

RT-PCR with primers designed to amplify the helicase domains of PvEV1, PvEV2 and PvEV3 was used to generate amplicons for Sanger sequencing. In this study, 26 common bean cultivars and landraces were investigated. PvEV1, PvEV2 and PvEV3 helicase sequences were identified in, respectively, ten, six and three common bean cultivars of the 26 tested (Table 1). Single infections of PvEV1 were identified in the four cultivars RED40, RWR1668, GLP1127 and G2333. PvEV1 and PvEV2 double infections were identified in the three cultivars KK022, SER16 and RWR2245. Combined infections of PvEV1, PvEV2 and PvEV3 were identified in lines KK072, RWR2075 and MCM2001. In the lines containing mixed infections, none harboured PvEV2 without also containing PvEV1, and no cultivar was infected with PvEV3 without containing both PvEV1 and PvEV2 (Table 1).

### Phylogenetic and pairwise identity analyses based on regions encoding helicase sequences revealed clear species demarcations

**Fig. 1:**
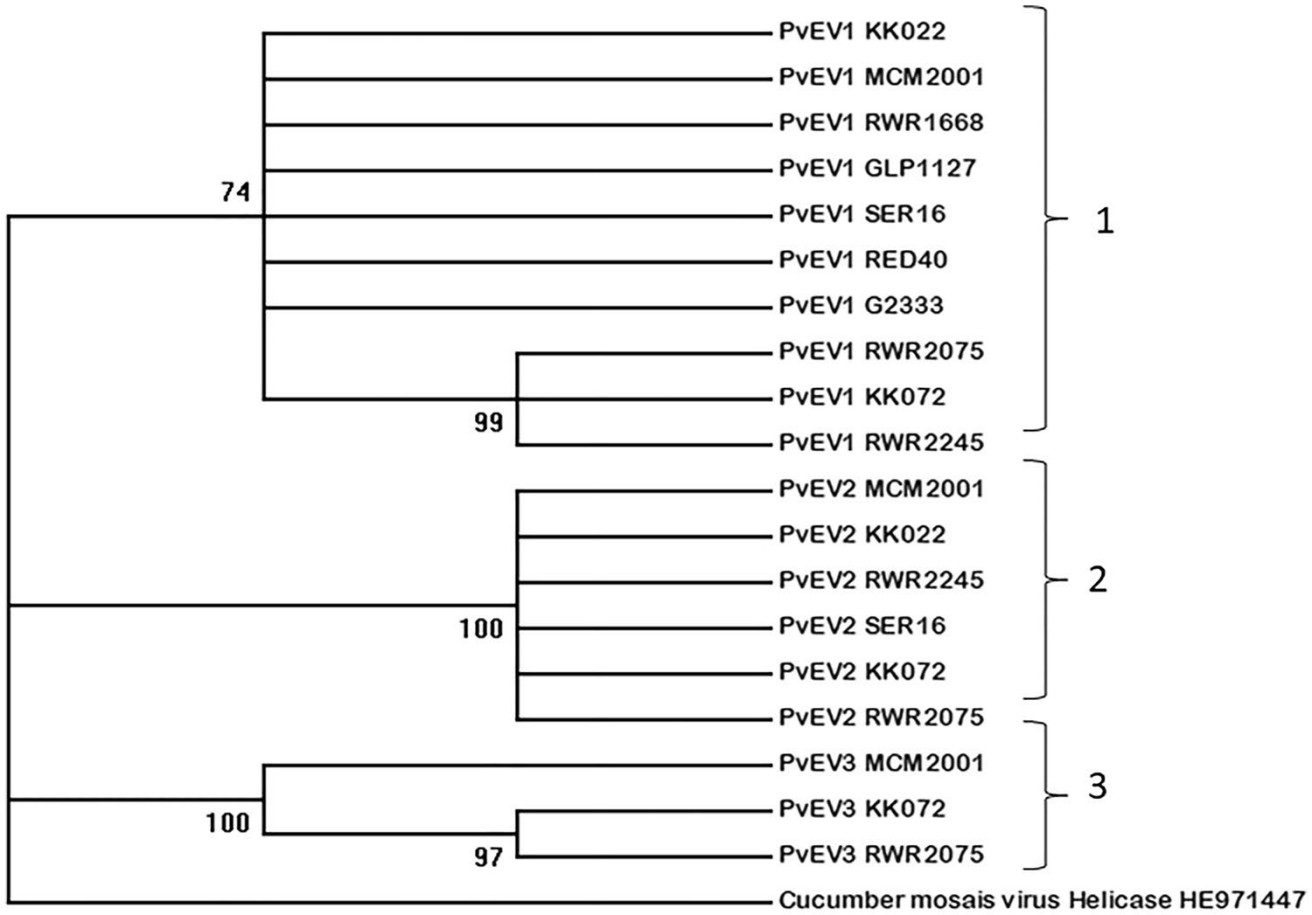
Phylogeny based on the sequences for the Helicase sequence domains of PvEV1, 2 and 3. Maximum-likelihood phylogenetic tree using Helicase domain nucleotide sequences from samples used in this study revealed three clades (1–3) demarcated according to species (PvEV1, PvEV2 and PvEV3). The branch structure is labelled with numbers indicating the percentage of bootstrap replicates supporting the outcome. Node significance was evaluated with 1000 bootstrap replicates. The GenBank accession number for the CMV Helicase sequence used as an outgroup is included in brackets

**Fig. 2:**
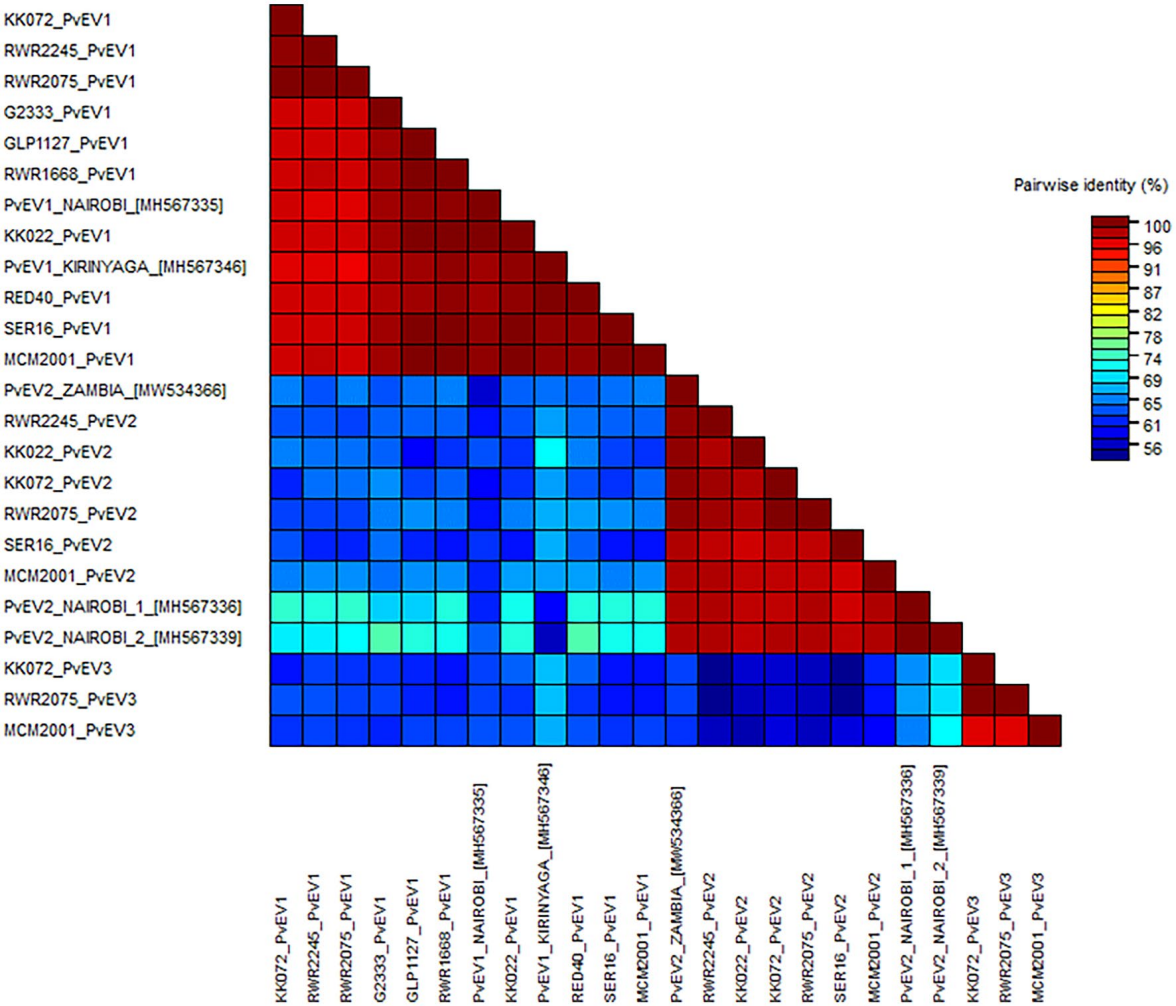
Pairwise sequence comparisons of PvEV1, 2 and 3 using sequences derived from the helicase region. Comparisons conducted in Sequence Demarcation Tool revealed clear demarcation between the three species. In this analysis, two comparator sequences (PvEV1 Nairobi MH567335 and PvEV1 Kirinyaga MH567348) previously identified as PvEV1 [31] and three PvEV2 sequences PvEV2 Nairobi 1 MH567336 and PvEV2 Nairobi 2 MH567339 [31] and PvEV2 Zambia MW534366 (downloaded from GenBank) were used to confirm the accuracy of the demarcation. Intraspecies sequence comparisons (Red) were PvEV1 95.7–100%, PvEV2 96.4–99.3%, and PvEV3 95.5–100%. Interspecies similarity (Blue) ranged between 54.4 and 64.7% when PvEV1 and PvEV2 were compared, 59.4–67.4% between PvEV1 and PvEV3 and 53.6–70.5% between PvEV2 and PvEV3

Phylogenetic analyses carried out using nucleotide sequences generated by Sanger sequencing of the helicase coding regions of our test varieties revealed three clades that corresponded to the endornaviruses PvEV1, PvEV2 and PvEV3 (Fig. 1). Further, pairwise comparisons were conducted using the Sequence Demarcation Tool. In this analysis, we downloaded from GenBank comparator nucleotide sequences for the helicase region for PvEV1 (GenBank accession numbers: MH567335 and MH567346) and PvEV2 (GenBank accession numbers: MW534366, MH567336.1 and MH567339.1). These sequences of known species identity were used to confirm the accuracy of the sequence demarcation analyses. We did not include any helicase sequences for PvEV3 from GenBank as there were none available. We observed for intraspecies nucleotide-level comparisons, our PvEV1 sequences were 95.7–100% similar to the comparator sequences from GenBank. Comparatively, PvEV2 had 96.4–99.3% similarity with the comparator sequences from GenBank (Fig. 2, Table S2). For inter-species sequence comparisons, the nucleotide-level similarity ranged from 54.4 to 64.7% between PvEV1 and PvEV2; 59.4–67 between PvEV1 and PvEV3 and 53.6–70.5% between PvEV2 and PvEV3 (Fig. 2, Table S2). The results confirmed clear sequence demarcation even when the sequences had been derived from varieties containing mixed endornavirus infections.

### High-throughput sequencing of the bean cultivars KK022 and KK072 revealed near-complete genomes of PvEV1, PvEV2 and PvEV3

The bean cultivars KK022 and KK072, shown by RT-PCR to be, doubly infected with PvEV1 and PvEV2 and triply infected with PvEV1, PvEV2 and PvEV3, respectively, were chosen for further analysis by HTS. The sequences derived from HTS using Illumina NextSeq were analysed with VirFind for de novo assembly of contigs. Multiple contigs were assembled from the KK022 sequences and were taxonomically annotated as either PvEV1 or PvEV2. Results for KK072 showed multiple contigs annotated as PvEV1, PvEV2, and PvEV3 (Table S3). These results were consistent with our findings by RT-PCR for these samples.

The contigs with sequence similarity to PvEVs were mapped to reference genomes and five consensus sequences were generated. The genomes of both PvEV1 isolates (from samples KK022 and KK072) were 14,071 nucleotides long which was one nucleotide shorter than the reference genome. The assembled genomes of PvEV2 from KK022 and KK072 were 14,820 nucleotides in length which was comparable in length to the reference sequence. The genome of the sequenced isolate of PvEV3 spanned 15,204 nucleotides and was one nucleotide shorter than the reference genome. Although 3′–5′ RACE was not carried out, we considered our sequences near-complete genomes given that their lengths were comparable to those in GenBank. The sequences were submitted to GenBank for the assignment of accession numbers (Table S4).

### Sequence analyses and phylogenies of PvEV1, PvEV2 and PvEV3

**Fig. 3:**
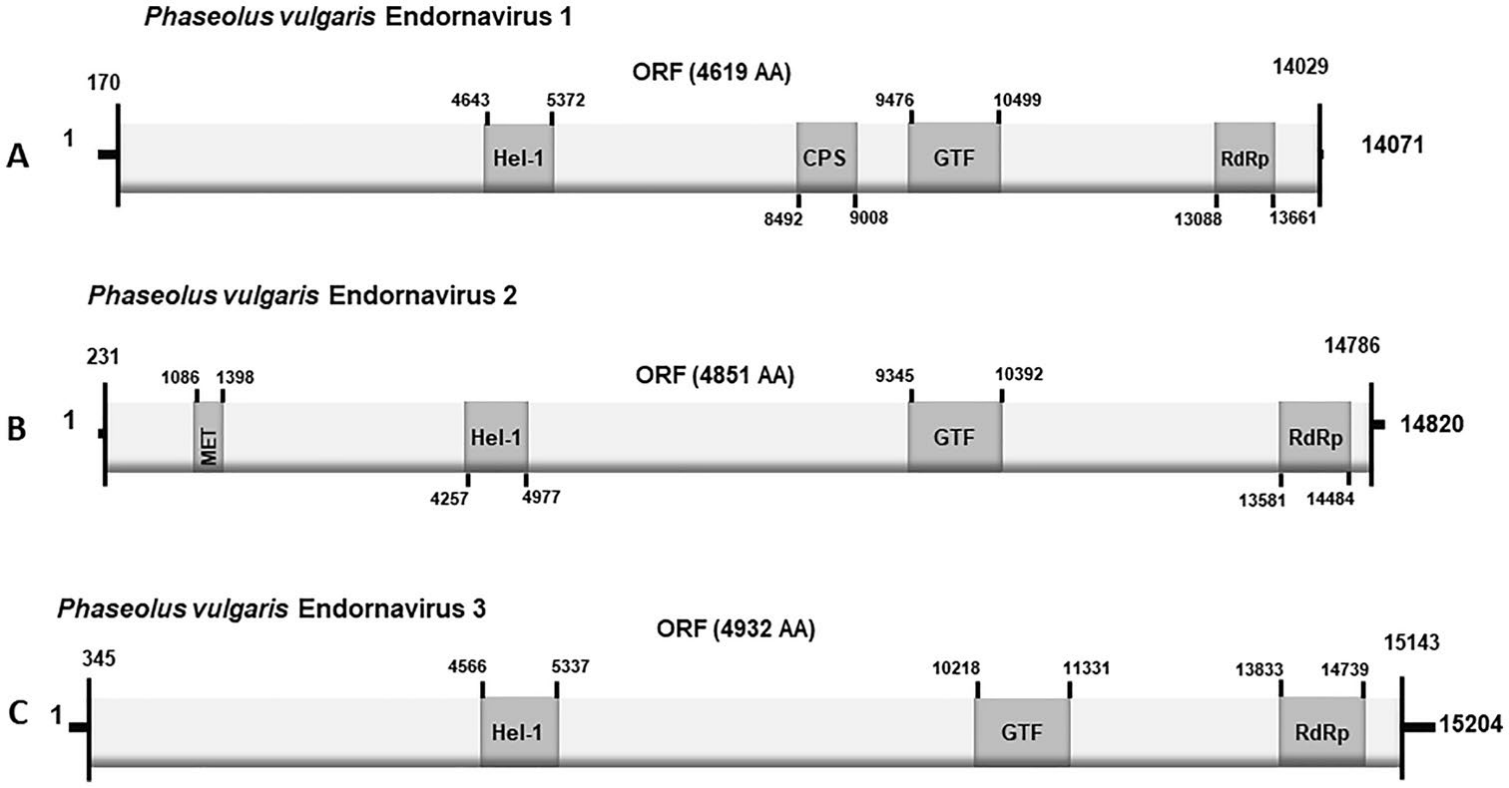
Inferred genome organization of *Phaseolus* *vulgaris* endornavirus (PvEV)1, PvEV2 and PvEV3 isolates detected in this study. The boxes (**A**–**C**) represent the three endornaviruses detected in this study. Key nucleotide references for the 3′ and 5′ untranslated regions are listed, and the predicted positions for the domains are identified. The lengths of the three species were 14,071 (PvEV1), 14,820 (PvEV2) and 15,204 (PvEV3) nucleotides long. These sequences, individually translated into protein, formed a single open reading frame (ORF). The lengths of the ORFs are indicated in parentheses above each box. The nucleotide positions from which the ORFs begin and end are indicated above the solid black bars at the ends of each box. The horizontal lines at each box 3′ and 5′ represent the untranslated regions (UTRs). The dark grey boxes with nucleotide positions indicated above short vertical lines within the larger box represent where the putative domains were identified in the Conserved Domain database. *Hel-1* Helicase, *CPS* capsular polysaccharide synthase, *GTF* glycosyltransferase, *MET* methyltransferase, *RdRp* RNA-dependent RNA polymerase

For this analysis, we conducted a comparison of nucleotide sequences between the consensus sequences and the reference sequence used for mapping. The near-complete genome sequences of PvEV1 KK022 and KK072 exhibited 97.98% and 94.69% similarity, respectively, with the PvEV1 sequence (MF375892) from GenBank. Utilizing the ORF finder tool and the near-complete genome sequences, we determined that sequences from both KK022 and KK072 encoded a single putative polyprotein, consistent with other PvEV1 genomes available in GenBank. However, there was a disparity in the predicted ORF length between our sequences and the reference genome. Whilst the reference genome’s ORF consisted of 4618 amino acids, our sequences exhibited an ORF of 4619 amino acids, with an additional serine residue at position 3499. When comparing the ORF sequences of PvEV1 KK022 and KK072 with the protein sequence AVD68677 from GenBank derived from the reference sequence we used, we observed amino acid-level similarities of 98.55% and 96.38%, respectively. Similar to other PvEV1 genomes in GenBank, our sequences possessed four conserved domains. These included a helicase domain (nucleotides 4643 to 5372), a domain resembling a bacterial capsular polysaccharide synthase (CPS) (nucleotides 8492 to 9008), a glycosyltransferase domain (nucleotides 9476 to 10499) and an RdRp domain near the 5′ end (nucleotides 13088 to 13661) of the assembled genomic RNA sequence (Fig. 3a).

The consensus sequences of PvEV2 KK022 and KK072 had 98.27% and 99.03% nucleotide-level similarity with sequence PvEV2 sequence AB719398 from GenBank. Using the ORF Finder tool in GenBank, we determined that the sequence for PvEV2 ORF encoded a putative polyprotein of 4920 amino acids. This was similar in length to that of isolate KT456288 in GenBank. However, compared to the reference genome (AB719398), the ORF we identified was 69 aa longer. On analysing the reference sequence, we ascertained that the ‘additional’ 69 aa was present when the nucleotide sequence of isolate AB719398 was virtually translated into a polyprotein in the same software. However, the authors truncated the ORF from the second ‘AUG’ (methionine), which resulted in a polyprotein 4851 aa long, similar with earlier convention in published sequences [14, 43]. Therefore, in keeping with this convention, we annotated similarly (Fig. 3b). The ORF protein sequence PvEV2 KK022 and KK072 had 98.52% and 99.28% amino acid-level similarity with sequence PvEV2 sequence BAM68540.1 from GenBank derived from the reference sequence we used. In further analyses, we observed that the putative polyprotein of our PvEV2 sequences contained a methyltransferase domain encoded by nucleotides at positions 1086 to 1398, as well as a helicase domain (encoded by nucleotides at position 4257–4977), a glycosyltransferase domain (encoded by nucleotides at position 9345–10392), and an RdRp domain at the 5′ end (encoded by nucleotides at positions 13833–14484) (Fig. 3b). These findings were similar to those reported in other sequences in GenBank, such as AB719398, which we used in our analyses.

The consensus sequence of PvEV3 had 93% similarity with PvEV3 isolate NC_040558 from GenBank. The protein sequence of the PvEV3 ORF had 95.32% similarity with sequence PvEV3 sequence YP_009551959.1 from GenBank derived from the reference sequence we used. Our polyprotein sequence was 4932 aa, similar to the one reported in GenBank (accession number NC_040558) (Fig. 3c). Three potential functional domains were identified: helicase (encoded by nucleotides 4566–5337), glycosyltransferase (encoded by nucleotides 10218–11331), and RdRp (encoded by nucleotides 13833–14739) (Fig. 3c).

**Fig. 4:**
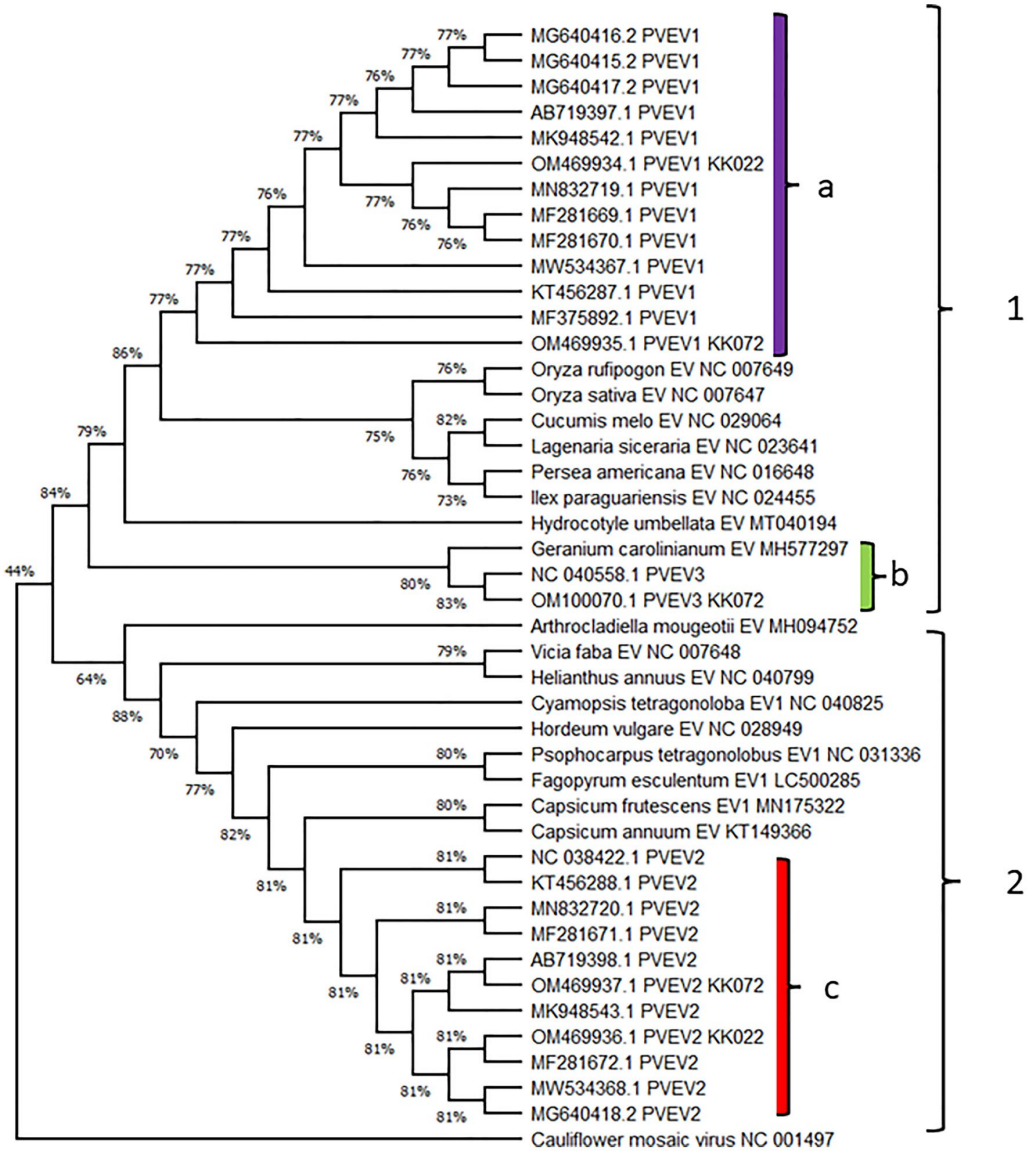
Whole-genome phylogeny of PvEV1, 2 and 3 and Endornaviruses from other plant hosts. Two major clades (‘1’ and ‘2’) were revealed by phylogenetic analysis using the Maximum Likelihood method. PvEV1 (‘a’; purple bar) and 3 (‘b’; green bar) were in a separate clade from PvEV2 (‘c’; red bar). Whilst PvEV1 sequences clustered on their own in their subclade, PvEV3 sequences shared a subclade with an endornavirus sequence isolated from *Geranium carolinianum.* PvEV2 shared a subclade closest to endornaviruses found in wild and domesticated chili pepper (*Capsicum frutescens* and *Capsicum annuum*). The branch structure is labelled with the percentage of bootstrap replicates supporting the outcome. Node significance was evaluated with 1000 bootstrap replicates

The phylogenetic analyses conducted using complete and near-complete genome sequences of PvEV1, PvEV2 and PvEV3 from GenBank, including sequences from our study and comparator sequences from other plant species, revealed distinct patterns. Two primary clades were identified, with PvEV1 and PvEV3 forming a separate clade from PvEV2. Specifically, PvEV1 sequences formed a distinct subclade, whilst PvEV3 clustered within a subclade that also included a sequence detected in *Geranium carolinianum*. PvEV2, on the other hand, formed a separate clade, with its sequences clustering together (Fig. 4). Notably, these clades demonstrated clear demarcation based on the respective host plants.

## Discussion

Two main forms of common bean (*P. vulgaris*) are grown around the world: the snap bean (green pods harvested), and the dry bean (seeds harvested). In sub-Saharan Africa, dry beans (also referred to as grain) account for 95% of beans grown and are used for local consumption, whilst the snap bean variety, a horticultural crop accounts for 3% and is grown mainly for export [44]. The remaining 2% is Tepary bean (*Phaseolus acutifolius*) which is localized in different parts of sub-Saharan Africa [44]. Consequently, the region is highly dependent on dry bean cultivation for its protein needs [25]. Most of the common bean varieties used in our study are primarily grown for grain and seed by smallholder farmers with less than 5 acres of land. An earlier metagenomic study had detected the presence of endornaviruses (PvEV1 and PvEV2) in common bean plants sampled in smallholder farms in peri-urban and rural Kenyan fields [31]. However, that study did not identify the specific common bean varieties harbouring these Endornaviruses.

Our study systematically screened common bean varieties from the east African region to determine if endornaviruses were widespread in popular lines. Using RT-PCR, we detected all three endornaviruses. A key finding was the first detection of PvEV3 in African-grown lines. PvEV3 had previously only been identified in a few North American cultivars [12]. The occurrence of endornaviruses was not linked to geographical location, and it is therefore likely that in East Africa, bean endornaviruses are spread by imports of germplasm from elsewhere and not due to local varietal preferences or inadvertent selection by local plant breeders.

We observed single infections of PvEV1 in six varieties. However, we did not observe any single infections of PvEV2, which was consistent with results reported earlier by Okada and colleagues when they screened 50 bean varieties, including wild relatives, in their seminal report on the detection of PvEV3 [12]. We did not observe any single infection of PvEV3, which contrasts with the findings of Okada and colleagues, who observed single PvEV3 infection in two domesticated varieties of bean, ‘Clouseau’ and ‘PI 209488’ of Andean and Mesoamerican origins, respectively, and in four wild bean varieties, all of Mesoamerican origin [12].

We observed mixed infection in six bean varieties. KK022, RWR2245 and SER16 had double infection with PvEV1 and PvEV2 and another three varieties, KK072, MCM 2001 and RWR 2073 of Kenyan, Ugandan and Rwandan provenance, respectively, had mixed infections of all three endornaviruses. Okada and colleagues have reported mixed infections, either double or triple, for endornaviruses [12]. We did not observe any double infections involving either PvEV1–PvEV3 or PvEV2–PvEV3 combinations. This was different from the findings of Okada and colleagues, who reported detecting PvEV2–PvEV3 infection in variety ‘Red Rover’, an Andean variety and PvEV1–PvEV3 infection in two landraces (W6 12107 and PI 309885) of Mesoamerican origins [12]. Our screening of 26 popular varieties makes for a modest start. Should screening other bean varieties within the East Africa region for endornaviruses be required, we may likely encounter similar results. According to the Pan African Bean Research Alliance, a bean research network active in 32 African countries, over 400 improved varieties adapted to different agroecological zones have been released for use in sub-Saharan Africa [44].

RNAseq analysis performed using near-isogenic lines of the cultivar Black Turtle Soup found 132 genes differentially expressed between plants doubly infected with PvEV1 and PvEV2 compared with control plants that were not infected with either of the endornaviruses [45]. Plants harbouring PvEV1 and PvEV2 yielded faster germinating seeds that gave rise to seedlings with longer radicles, had a lower chlorophyll content, but higher carotene content, produced longer pods, and yielded seeds with a greater average mass than control plants [20]. It is possible that the effects on gene expression [45] explain many, if not all, of the changes in plant physiology [20]. Arguably some of these virus-associated traits are beneficial to the plants and could be agronomically valuable. However, some traits conferred by other PVs, such as the amalgavirus STV, which appears to increase susceptibility to infection by other viruses [22], are not. 

Using high-throughput sequencing and mapping to consensus sequences, we generated near-complete genomes for the three endornaviruses. Okada and colleagues [14] reported the length for PvEV1 to be 13,908 nucleotides and that for PvEV2 as 14,820 nucleotides. The sequences we generated were longer for PvEV1 (14,071 nucleotides) but similar for PvEV2 (14,820 nucleotides). Meanwhile, our PvEV3 genome length at 15,204 nucleotides was shorter than that reported by Okada and colleagues [12] by a single nucleotide in length (15,205 nucleotides). These differences in length could be attributed to the methods used to generate the genome information. Pairwise comparisons of nucleotide and protein sequences showed that our reported sequences had sufficient inter-species differences for definitive sequence demarcation along species lines. As noted by the ICTV, in outlining the species demarcation criteria in the genus, members of different species have an overall nucleotide sequence identity below 75% [6]. Phylogenetic analyses of the whole genomes and helicase regions further evidenced this.

Common bean is susceptible to a range of plant pathogens, including viruses [28, 46–48]. Breeding for resistance has offered protection against some viral diseases. For example, the *I* gene protects against some strains of bean common mosaic virus, although it renders plants carrying this dominant resistance allele vulnerable to systemic necrosis induction by bean common mosaic necrosis virus [28]. However, for some pathogenic viruses detected in bean, such as CMV [31], there is no effective genetic resistance. Therefore, tools that enhance protection must be found. As noted earlier, research into endornaviruses shows a mixed record of what their presence can cause to plants [18]. Therefore, depending on whether or not these inherited viruses are considered beneficial, their presence could be helpful for bean breeders to ensure or avoid the incorporation of endornaviruses or other PVs into new plant lines. Future studies should determine whether bean endornaviruses confer advantages to the host plant.

## Supplementary Information

Below is the link to the electronic supplementary material.

Supplementary file1 (XLSX 113 KB)

## Data Availability

All near-complete genome sequences described in this report are uploaded to GenBank. However, we have also made these sequences, their ORF sequences and Helicase sequences used on phylogeny and pairwise comparisons available as supplementary information (Table S5). The dataset in this study is available in the Sequence Read Archive (SRA) repository with accession numbers PRJNA885619.
